# Multimodal Magnetic, Photothermal, Ultrasonic, and Vibrational Actuation of Drug-Loaded Superparamagnetic Iron Oxide Nanoparticles for Enhanced Transport Across Semipermeable Membranes

**DOI:** 10.3390/bioengineering13070834

**Published:** 2026-07-21

**Authors:** Thiraj Mohankumar, Veil Denise Plazuela, Sergey Budko, Daniel Quain Sun, Donglu Shi

**Affiliations:** 1 Department of Mechanical and Materials Engineering, University of Cincinnati, Cincinnati, OH 45221, USA; mohanktd@mail.uc.edu (T.M.); plazueva@mail.uc.edu (V.D.P.); 2 Ames Laboratory, Iowa State University, Ames, IA 50011, USA; 3 Department of Otolaryngology—Head and Neck Surgery, University of Cincinnati College of Medicine, Cincinnati, OH 45267, USA; 4 Department of Biomedical Engineering, University of Cincinnati, Cincinnati, OH 45221, USA

**Keywords:** hearing loss treatment, round window membrane, drug delivery, transport, magnetic nanoparticles, photothermal heating, mechanical vibration

## Abstract

The round window membrane (RWM) presents a major barrier to local drug delivery into the inner ear. Although magnetically guided superparamagnetic iron oxide nanoparticles (SPIONs) have shown promise for enhancing transport across the RWM, the effectiveness of magnetic-field-driven delivery decreases rapidly with distance from the magnet, limiting clinical applicability. In this study, PEGylated SPIONs were investigated as externally actuated carriers for enhanced transport across membrane barriers using magnetic, photothermal, ultrasonic, and vibrational stimulation. Nanoparticle transport was evaluated using a custom dual-chamber benchtop platform containing porcine small intestinal submucosa (SIS) membranes as a model transport barrier. Transport studies demonstrated that magnetic-field-assisted delivery significantly increased magnetic nanoparticle (MNP) transport rates relative to passive diffusion; however, transport enhancement decreased sharply with increasing magnet-to-membrane distance. To overcome this limitation, alternative external actuation strategies were explored. Laser-induced photothermal heating, ultrasonication, and mechanical vibration all significantly enhanced MNP transport, even in the absence of magnetic fields. Among the conditions examined, combined magnetic and photothermal stimulation produced the highest transport rates, indicating synergistic enhancement. These results show that MNP transport can be effectively enhanced through magnetic, thermal, and mechanical mechanisms. The findings establish a multimodal transport-engineering framework for improving drug delivery across the RWM and suggest clinically translatable alternatives to magnetic-field-only approaches for inner-ear therapy.

## 1. Introduction

Localized drug delivery to the inner ear remains limited by transport across the round window membrane (RWM). Although intratympanic administration provides an attractive non-systemic route, therapeutic efficacy depends on the ability to move drugs and carrier systems from the middle ear into the cochlea at sufficient concentrations [1,2]. This challenge is particularly important for nanoparticle-based carriers that do not rely solely on passive diffusion [2,3,4]. Consequently, successful cochlear delivery requires not only appropriate therapeutic payloads but also engineered transport vehicles capable of enhancing transfer across the RWM [5,6,7]. From a biomedical engineering perspective, the RWM functions as a multilayer transport barrier separating donor and receiver compartments [3,8,9,10]. Its structure, thickness, and cellular organization impose substantial resistance to mass transfer, making it both the primary access route to the inner ear and a major obstacle to therapeutic delivery [11,12,13,14]. This transport resistance often limits intracochlear drug concentrations despite direct placement of therapeutics within the middle ear [9]. These physiological constraints make model selection an engineering as well as a biological issue, because in vivo perilymph sampling and simplified in vitro membrane constructs do not fully capture the transport behavior of intact native RWM tissue [15,16,17]. In contrast, benchtop and ex vivo platforms preserve native ECM while allowing controlled variation in carrier properties and external forcing inputs, and staged progression from acellular semiporous membranes to native explants provides a practical framework for isolating the effects of particle size, surface chemistry, membrane permeability, and external actuation before testing under more physiologically relevant conditions [8,16,17,18].

Nanoparticle carriers provide a versatile platform for addressing this challenge because transport-relevant properties including size, surface charge, coating architecture, colloidal stability, and payload incorporation can be engineered [19,20,21,22,23,24,25]. Among available systems, PEGylated superparamagnetic iron oxide nanoparticles (SPIONs) are particularly attractive because they combine tunable surface chemistry with external actuation capability [26,27,28,29,30,31,32]. The Fe_3_O_4_ core provides magnetic responsiveness, while PEG coatings improve aqueous stability and biocompatibility. Previous studies have demonstrated that magnetically guided SPIONs can enhance transport across the RWM, with external magnetic fields increasing nanoparticle delivery by approximately 45–50% in guinea pig explant models [9,10]. These findings established a proof of concept for magnetic nanoparticle-mediated cochlear delivery. Despite these advances, magnetic-field-assisted transport faces a fundamental limitation. The magnetic field generated by a permanent magnet decreases approximately as 1/r^3^ with distance, while the corresponding field gradient responsible for nanoparticle actuation decreases even more rapidly [33,34,35]. As a result, magnetic transport efficiency diminishes substantially as the separation between the magnet and target tissue increases, creating challenges for clinical translation where the cochlea is located several centimeters beneath the skin surface.

This limitation motivates investigation of complementary transport-enhancement strategies. In addition to their superparamagnetic properties, Fe_3_O_4_ nanoparticles exhibit photothermal behavior that enables localized heat generation under laser irradiation. Thermal activation may increase diffusion rates and reduce transport resistance at the membrane interface [36,37,38]. Similarly, mechanical vibration and ultrasonication can enhance transport through microstreaming, convective mixing, transient membrane deformation, and boundary-layer disruption [39,40]. By combining magnetic guidance with thermal and mechanical stimulation, a multimodal transport platform may overcome the distance-dependent limitations of magnetic actuation alone. Beyond external driving forces, nanoparticle transport across the RWM is influenced by complex membrane interactions and active cellular processes, including macropinocytosis and caveolin-mediated endocytosis [1,16]. Previous studies using steroid-loaded magnetic nanoparticles have further demonstrated the therapeutic potential of externally actuated delivery systems in hearing-loss models [6,26]. Together, these findings suggest that transport performance depends on the coupled effects of nanoparticle design, membrane properties, and applied physical stimuli.

In this study, we investigate PEGylated SPIONs as externally actuated carriers for enhanced transport across membrane barriers relevant to cochlear drug delivery. Transport was first evaluated using an acellular porcine small intestinal submucosa (SIS) membrane model to establish a controlled baseline for passive and field-driven transport. A custom dual-chamber benchtop platform was used to compare passive diffusion magnetic, photothermal, ultrasonic, and vibrational actuation, as well as selected multimodal combinations. Dexamethasone-conjugated nanoparticles were incorporated as a translational payload. We hypothesize that externally actuated PEGylated SPIONs will exhibit mode-dependent transport enhancement, and that comparative evaluation of these actuation strategies will provide design principles for clinically viable inner-ear drug delivery systems.

## 2. Experimental Procedures

### 2.1. Nanoparticle Synthesis and Drug Loading

Iron oxide nanoparticles with a core size of approximately 9 nm were synthesized by thermal decomposition of iron (III) acetylacetonate (Fe(acac)_3_, 98%) in the organic phase. Briefly, 4 mmol (0.763 g) of Fe(acac)_3_ was mixed with 20 mL benzyl ether and 20 mL oleylamine. The mixture was heated at 110 °C for 1 h for dehydration, followed by reflux at 300 °C for 30 min. After cooling to room temperature, the precipitate was collected, washed three times with ethanol, and redispersed in hexanes. The nanoparticles were stabilized by the addition of approximately 0.25 mL oleic acid [9,21].

Surface modification was performed by replacing the oleylamine coating on the synthesized Fe_3_O_4_ nanoparticles with polyethylene glycol (PEG)-based ligands to generate water-dispersible nanoparticles with different coating thicknesses. α,ω-bis{2-[(3-carboxy-1-oxopropyl) amino] ethyl} polyethylene glycol ($M_{r}$ = 3000, Sigma-Aldrich, St Louis, MO, USA), Amine-PEG-acid (COOH-PEG-NH_2_; $M_{w}$ = 1 k and 3.4 k, BioPharmaPEG, Watertown, MA, USA) were used and differentially grafted to Fe_3_O_4_ monocores to generate thin and thick surface coatings. In a typical reaction, 20 mg of α,ω-bis{2-[(3-carboxy-1-oxopropyl) amino] ethyl} or polyethylene glycol Amine-PEG-acid were used to prepare PEGylated iron nanoparticles. These were mixed with 2 mg N-hydroxy succinimide (NHS), 3 mg 1-ethyl-3-(3-dimethylaminopropyl) carbodiimide (EDC), 1.27 mg dopamine hydrochloride, 10 mg Na_2_CO_3_, 1 mL chloroform (CHCl_3_) and 1 mL dimethylformamide (DMF), and stirred under nitrogen protection for 4 h. Subsequently, 5 mg of Fe_3_O_4_ nanoparticles dissolved in 1 mL chloroform were added to the reaction mixture and allowed to stir overnight (~12–14 h) under nitrogen protection. The particles were collected using hexanes and separated by centrifugation at 4000 rpm for 60 min. The supernatant was removed, and the particles were briefly dried to remove residual organic solvent. The nanoparticles were then dispersed in 5 mL phosphate-buffered saline (PBS) followed by sonication for 20–30 min to ensure complete aqueous dispersion. Residual reactants and unbound chemicals were removed by dialysis. For this step, the nanoparticle suspension was loaded into a 12 mL Slide-A-Lyzer dialysis cassette with a 10 kDa MWCO (Thermo Fisher Scientific, Waltham, MA, USA) and dialyzed against deionized water for 24 h under stirring at 500 rpm [9,10,21].

Dexamethasone conjugation was carried out through succinate derivatization followed by EDC/NHS-mediated amide coupling [41,42]. Briefly, dexamethasone reacted with succinic anhydride in the presence of DMAP in anhydrous organic solvent to yield dexamethasone hemi succinate, thereby introducing a terminal carboxyl group suitable for further coupling. The product was isolated and purified prior to conjugation using a rotary evaporator. Separately, Fe_3_O_4_ nanoparticles bearing terminal amine functionality were prepared as described above. Dexamethasone hemisuccinate was then activated with EDC and NHS and added to the amine-functional PEGylated nanoparticle suspension under stirring. The reaction was allowed to proceed for 4 h at room temperature, after which the conjugated nanoparticles were purified by dialysis using a 10 kDa MWCO cassette (Thermo Fisher Scientific, Waltham, MA, USA) to remove free dexamethasone and residual coupling reagents. The final particles were collected in PBS for subsequent characterization. Drug conjugation efficiency was determined by measuring the amount of unconjugated dexamethasone remaining in the supernatant and dialysis medium using LC-MS.

Physicochemical characterization was performed to evaluate the structural, interfacial, and functional properties of the PEGylated magnetic nanoparticle formulations prior to membrane transport studies and dexamethasone conjugation. These measurements were used to determine whether nanoparticle synthesis and PEGylation produced the intended changes in morphology, size distribution, surface charge, photothermal responsiveness, and colloidal stability. Because these parameters directly influence dispersion quality, carrier–membrane interaction, and actuation-assisted transport behavior, characterization was carried out systematically for each nanoparticle formulation before use in transport experiments.

### 2.2. Experimental Set up for Nanoparticle Transport

**Figure 1:**
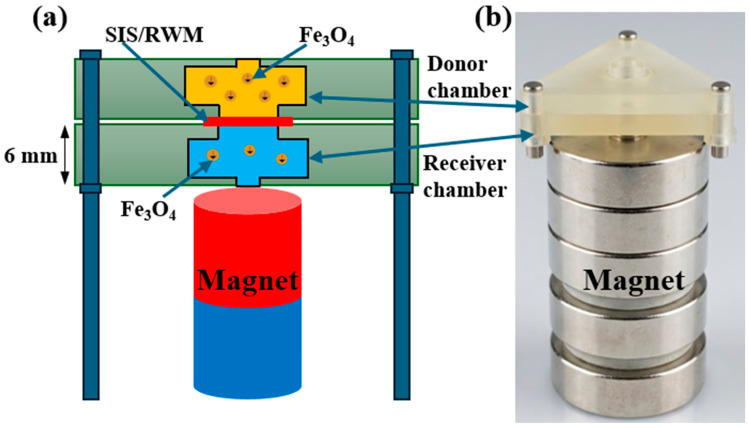
Dual-Chamber Membrane Transport System (DCMTS). (**a**) Schematic of DCMTS (donor/receiver, 400 μL each) used to quantify nanoparticle transport across semipermeable membranes under controlled conditions. (**b**) Photograph showing a stack of cylindrical neodymium magnets (1.5 × 3 inch, Grade N42) positioned adjacent to the membrane to generate a magnetic field gradient normal to the transport interface. Porcine SIS was mounted between chambers, with PEGylated SPIONs loaded in the donor chamber and PBS in the receiver chamber under sink conditions. Transport was assessed under passive and externally actuated modes, including magnetic, photothermal, vibrational, and ultrasonic stimulation.

A custom Dual-Chamber Membrane Transport System (DCMTS) was fabricated by 3D printing to provide a controlled benchtop platform for evaluating nanoparticle transport across semi-permeable membrane barriers (Figure 1a). The device consisted of donor and receiver chambers (400 μL each) separated by a membrane insert, enabling quantitative assessment of nanoparticle transport under externally applied physical stimuli. For magnetic actuation studies, a cylindrical permanent magnet was positioned adjacent to the membrane compartment to generate a magnetic field gradient across the transport interface (Figure 1b). Additional actuation modalities, including laser-induced photothermal stimulation, mechanical vibration, and ultrasonication, were incorporated to investigate their individual and combined effects on nanoparticle transport.

A membrane-based benchtop transport platform was used to evaluate nanoparticle passage under controlled donor–receiver conditions. As an initial model, an acellular porcine SIS membrane was used to establish baseline passive and actuation-assisted transport behavior before introducing the additional structural and biological complexity of native tissue. This approach enabled transport measurements to be performed in a simplified semiporous barrier system while maintaining a defined experimental geometry for comparative analysis. Similar benchtop membrane systems were used to study transport across biological barriers under controlled conditions [4,9,14]. Acellular porcine SIS membrane was selected as the initial transport barrier for baseline studies. The SIS membrane provided a cell-free semiporous interface for evaluating nanoparticle transport without the confounding effects of active cellular uptake, tissue viability, or native membrane heterogeneity. The SIS model was not intended to fully reproduce native RWM physiology, but rather to establish baseline transport behavior before validation in more physiologically complex RWM tissue. This simplified platform was used to compare transport behavior across passive and externally actuated conditions prior to subsequent validation in more physiologically relevant membrane models. Membranes were cut to the required dimensions, mounted between donor and receiver compartments, and sealed to prevent leakage during transport experiments.

Magnetic actuation was performed using a cylindrical neodymium magnet (1.5 × 3-inch, Grade N42, Applied Magnetics, Plano, TX, USA). The magnet generates a surface magnetic flux density on the order of 0.5–0.6 T at the pole face, with the field decreasing rapidly with distance according to a dipole-like decay behavior. At distances relevant to the membrane interface (6–8 mm), the estimated magnetic field strength is approximately 0.4 T depending on positioning relative to the central axis. For transport experiments, the cylindrical magnet was positioned with its axial direction perpendicular to the membrane plane, such that the flat pole face was oriented parallel to the membrane surface. This configuration maximized the magnetic field gradient normal to the transport interface, thereby promoting nanoparticle migration toward the membrane. The resulting spatially non-uniform magnetic field provides the driving force for nanoparticle migration toward the membrane surface with field-assisted transport diminishing with increasing separation from the magnet, consistent with classical magnetostatic scaling behavior.

Porcine SIS membranes were mounted between the donor and receiver chambers using silicone elastomer and epoxy sealants to establish a leak-free transport barrier. In selected experiments, freshly harvested guinea pig RWM specimens were similarly mounted to evaluate transport under physiologically relevant conditions. The donor chamber was loaded with PEGylated SPION suspensions in phosphate-buffered saline (PBS), while the receiver chamber contained fresh PBS. At predetermined time points (0.5, 1, 1.5, 2, and 4 h), the entire receiver-chamber volume was collected for analysis and replaced with fresh PBS to maintain sink conditions. Receiver-chamber samples collected during transport studies were analyzed using a Ferene-S spectrophotometric assay to quantify iron as a measure of transported Fe_3_O_4_ nanoparticle content [43]. Transport experiments were conducted under passive diffusion, magnetic actuation, photothermal stimulation, vibrational excitation, ultrasonication, and selected multimodal combinations to compare the relative effectiveness of each enhancement strategy. All studies were performed at room temperature unless otherwise specified. Passive nanoparticle transport without external actuation was used as the primary baseline for each formulation. Nanoparticle transport was quantified using the Ferene-S iron assay, which specifically measures transported iron oxide content. Membranes were visually inspected before and after transport experiments, and membranes showing visible defects or abnormal leakage behavior were excluded from analysis.

All quantitative transport experiments were performed using independent membrane/chamber assemblies as experimental replicates. Data are presented as mean ± standard deviation (SD) unless otherwise stated. Sample size was selected based on pilot transport experiments and prior benchtop membrane transport studies using similar chamber-based nanoparticle delivery platforms. Because this study was designed as a comparative transport-engineering study rather than an in vivo efficacy study, the sample size was intended to evaluate reproducible differences among actuation conditions.

**Figure 2:**
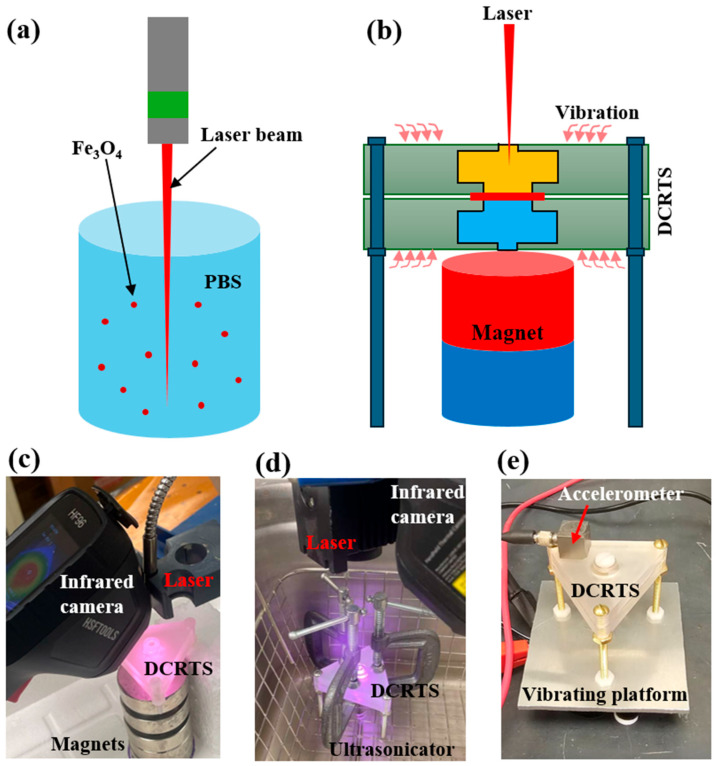
Multimodal actuation strategies for enhanced nanoparticle transport across membrane barriers. Schematic illustration of the experimental configurations used to evaluate externally stimulated transport of Fe_3_O_4_-based nanoparticles across semipermeable membranes. (**a**) Photothermal response of nanoparticle suspensions under 808 nm laser irradiation in solution, used to characterize intrinsic thermal conversion behavior. (**b**) Multimodal transport configuration in which laser irradiation is directed from above the donor chamber to induce localized heating and enhance transmembrane transport and Ultrasonication/Vibration was given to the chamber. (**c**) Magnetic assisted photothermal transport configuration in which laser irradiation is directed from above the donor chamber to induce localized heating and enhance transmembrane transport. (**d**) Ultrasound-assisted photothermal transport setup in which the entire device is immersed in an ultrasonic bath to deliver acoustic energy to the membrane interface. (**e**) Vibration-assisted photothermal transport configuration in which the transport device is mounted on a mechanical vibration platform to generate controlled oscillatory motion normal to the membrane surface. Across all conditions, membrane type, chamber geometry, nanoparticle concentration, fluid volumes, and sampling protocols were held constant to enable direct comparison of passive and actuation-enhanced transport modes.

Figure 2 shows the diagrams of multimodal actuation-assisted transport experiments across membrane interfaces. Following membrane mounting and loading of the donor and receiver chambers, transport experiments were conducted under passive and externally actuated conditions while maintaining identical baseline parameters, including membrane configuration, chamber geometry, nanoparticle concentration, fluid volumes, and sampling intervals. Passive transport, performed in the absence of any external stimulus, served as the reference condition for all comparative analyses. Figure 2a shows photothermal excitation in nanoparticle suspension. In solution-phase photothermal experiments, Fe_3_O_4_ nanoparticle suspensions were exposed to 785 nm laser irradiation to evaluate intrinsic photothermal conversion behavior and temperature rise under controlled optical stimulation. These measurements provided baseline thermal response characteristics of the nanoparticle system independent of membrane transport. Figure 2b shows the schematic of how all the transport assisted modes are incorporated into the transport chamber including photothermal and vibrational modes. Figure 2c shows the photothermal transport configuration with the magnets. For membrane transport studies, laser irradiation was directed from above the donor chamber toward the nanoparticle suspension, enabling localized photothermal heating within the donor compartment. The incident laser beam was aligned normal to the membrane interface to ensure spatially uniform energy delivery. Temperature evolution during irradiation was monitored using an infrared thermal imaging system to maintain reproducible thermal dosing conditions across experiments. Figure 2d shows the ultrasound-assisted transport set up. For acoustic stimulation experiments, the entire transport assembly was placed within an ultrasonic water bath system to provide uniform acoustic energy transmission to the membrane platform. The ultrasonic field was applied under controlled operational parameters, including frequency, power, and exposure duration, to evaluate the contribution of cavitation and acoustic streaming to nanoparticle transport enhancement. Figure 2e shows the vibration-assisted transport experimental set up. For mechanical stimulation experiments, the transport device was mounted on a programmable vibration platform positioned beneath the donor chamber. Controlled mechanical oscillations were applied normal to the membrane plane to induce dynamic fluid motion and boundary-layer disruption. Vibration frequency, amplitude, and duration were maintained consistently throughout each experimental run to enable quantitative comparison with other actuation modes. Across all actuation conditions, device orientation relative to the membrane interface was held constant, ensuring that observed differences in transport behavior could be attributed solely to the applied physical stimulus rather than geometric variability or baseline hydrodynamic effects.

Photothermal actuation was performed using 660, 808, 1064, and 1342 nm laser irradiation. The laser beam was aligned normal to the membrane plane and centered over the donor chamber/membrane region. Because the laser systems had different output powers and beam diameters at the sample surface, the temperature response was normalized to both laser power and beam area. The beam area was calculated from the approximate beam diameter at the sample surface. This normalization was used to compare the relative photothermal response among wavelengths despite differences in laser power and spot size. The time-dependent normalized temperature response is provided in Supplementary Information Figure S1, and the beam diameter, beam area, maximum temperature increase, and actuation geometry are summarized in Table S1.

## 3. Results

**Figure 3:**
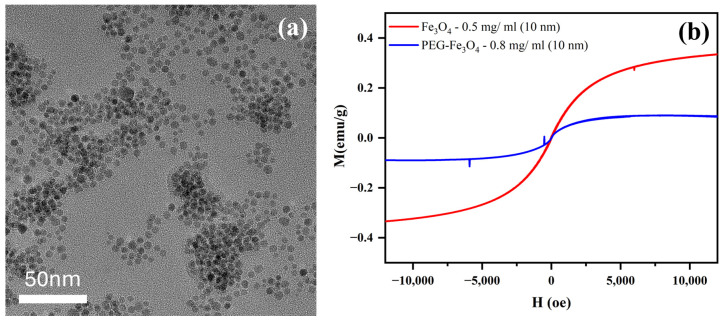
(**a**) Higher-Resolution TEM image showing Fe_3_O_0_ nanoparticles with diameters of approximately 10 nm. (**b**) Magnetization hysteresis curves of both PEGylated and as-synthesized Fe_3_O_4_ nanoparticles.

Transmission electron microscopy (TEM) analysis revealed that the synthesized Fe_3_O_4_ nanoparticles exhibited a relatively uniform morphology with narrow size distribution. As shown in Figure 3a, the as-synthesized nanoparticles are mono-dispersed with an average diameter of approximately 10 nm. The nanoparticles appeared well dispersed with minimal aggregation, indicating successful synthesis and stabilization. Figure 3b shows the magnetic hysteresis for as-synthesized Fe_3_O_4_ and PEGylated nanoparticles at the indicated concentrations. As shown in this figure, all nanoparticles exhibit superparamagnetic behaviors with reversible magnetic hysteresis. The PEGylated Fe_3_O_4_ nanoparticles retained strong magnetic responsiveness while exhibiting negligible remanence and coercivity, consistent with superparamagnetic behavior.

**Figure 4:**
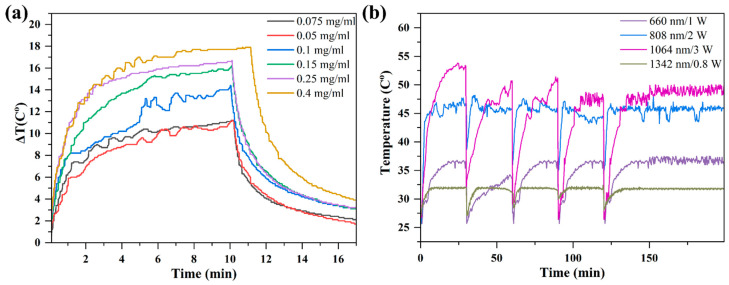
Photothermal heating and cooling behavior of PEGylated Fe_3_O_4_ nanoparticles under laser irradiation. (**a**) Temperature profiles of Fe_3_O_4_ nanoparticle suspensions at varying concentrations during irradiation with a 785 nm, 3 W laser. (**b**) Temperature profiles of Fe_3_O_4_ nanoparticle suspensions under laser irradiation at four different power settings. All samples exhibited rapid temperature increases upon irradiation followed by gradual stabilization as heat generation approached equilibrium with heat dissipation. Note that the lasers of different powers were turned on for a period of 25 min to observe the heating and cooling behaviors. Detailed actuation parameters, including laser beam diameter, maximum temperature rise, magnet orientation, ultrasound settings, and vibration calibration, are provided in Table S1. The normalized photothermal temperature response is shown in Figure S1.

Photothermal responsiveness of nanoparticles was evaluated by monitoring the temperature rise in suspensions and absorbance spectra of PEGylated magnetic nanoparticle platform prior to transport experiments. The samples were exposed to different wavelength laser light at variable power density and temperature was recorded as a function of time using an infrared camera. Figure 4a shows the heating and cooling profiles of Fe_3_O_4_ solutions irradiated with 785 nm/3 W laser with multiple concentrations. As shown in this figure, the temperatures of the solutions increase rapidly initially and level off after 5 min due to balance between the photothermal heating and heat loss. Laser was turned off at 10 min, resulting in sharp temperature falls. It is evident that the photothermal heating has a strong concentration dependence with the 0.4 mg/mL sample reaching the highest temperature.

For comparison of laser power effects with the nanoparticle transport experiments, Figure 4b presents the heating curves obtained using four different laser power settings. The laser was turned on and off for different time intervals (30 min, 60 min, 90 min and 120 min) to observe the heating and cooling behaviors and obtain continuous cumulative nanoparticle transport data.

In all cases, temperature increased with irradiation time and reached a plateau after several minutes of exposure. Higher laser powers produced faster heating rates and higher equilibrium temperatures, indicating that photothermal performance can be modulated by adjusting optical power density. These results confirm that the PEGylated Fe_3_O_4_ nanoparticle platform exhibits robust and controllable photothermal behavior, supporting its application in thermally assisted transport across membrane barriers. Additional wavelength-dependent studies showed that the photothermal response is dependent on the irradiation wavelength. Among the tested conditions, the strongest heating responses were observed under 808 nm and 1064 nm irradiation, whereas 1342 nm produced the lowest temperature increase. Irradiation at 660 nm generated a measurable but more moderate heating effect. Together, these findings show that both laser wavelength and power density influence nanoparticle-mediated photothermal heating and provide important design parameters for optimizing thermally enhanced drug delivery.

It should be noted that both 808 nm and 1064 nm laser irradiation produced plateau temperatures in the range of 45–50 °C. Although this temperature range is sufficiently high to promote photothermal enhancement of transport, it can be controlled below levels expected to cause significant thermal degradation of the NT3 by adjusting laser power.

**Figure 5:**
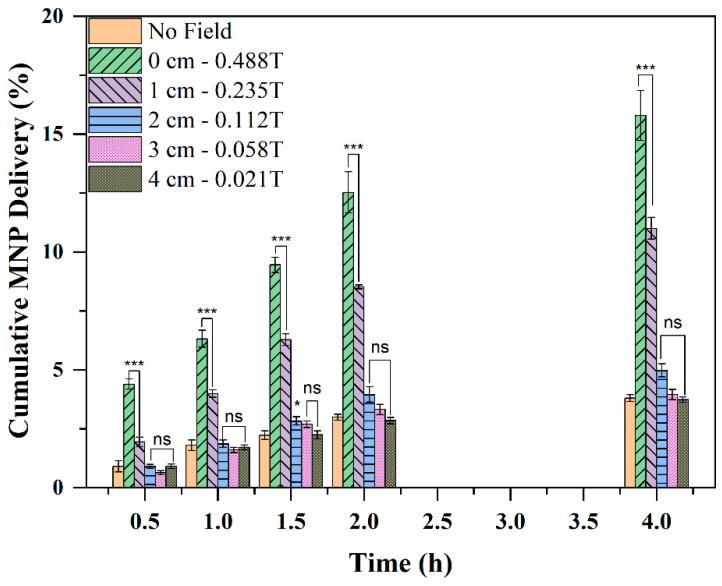
Effect of magnet-to-membrane distance on magnetic nanoparticle transport across the SIS membrane. Cumulative MNP Delivery Percentage (CMDP) of Fe_3_O_4_ nanoparticles across the SIS membrane as a function of time at different magnet-to-membrane separation distances. CMDP enhancement was greatest when the magnet was positioned closest to the membrane (0 cm gap) and decreased progressively with increasing distance, reflecting the rapid decay of the magnetic field gradient and magnetic driving force. Data are presented as mean ± SD from *n* = 3 independent membrane/chamber assemblies per condition. Statistical significance at 4 h was determined using one-way ANOVA followed by Tukey’s post hoc test. * *p* < 0.05, ** *p* < 0.01, *** *p* < 0.001 compared with the no-magnet control; ns indicates not significant.

Passive transport experiments were first conducted to establish the baseline transmembrane passage of PEGylated Fe_3_O_4_ nanoparticles across the SIS membrane in the absence of external actuation (Figure 5). Under these conditions, the number of nanoparticles transported in the receiver chamber increased gradually with time and served as the reference for comparison with magnetic, photothermal, ultrasonic, and vibrational enhancement strategies. In Figure 5 shows the Cumulative MNP Delivery Percentage (CMDP) of Fe_3_O_4_ nanoparticles transported across the SIS membrane as a function of time and magnet-to-membrane separation distance. When the magnet was positioned directly beneath the receiver chamber (0 cm gap), CMDP increased steadily from approximately 4.38% at 0.5 h to about 15.79% after 4 h, demonstrating a significant enhancement relative to passive diffusion. As the separation distance increased, however, CMDP enhancement progressively diminished. At a gap of 2 cm, CMDP was substantially reduced, while at a 4 cm separation, CMDP approached values observed under passive conditions without magnetic assistance. Magnetic enhancement was strongly dependent on the distance between the magnet and the membrane chamber. At 4 h, the highest transport was observed at 0 cm, followed by 1 cm, while transport decreased substantially at greater distances. One-way ANOVA showed a significant effect of magnet distance on nanoparticle transport at 4 h. Tukey post hoc analysis showed that transport at 0 cm and 1 cm was significantly higher than the no-magnet control, whereas transport at distances of 2–6 cm was not significantly different from the no-magnet condition. These results indicate that magnetic actuation was most effective when the magnet was positioned close to the membrane, with significant enhancement maintained up to 1 cm under the tested conditions.

The strong distance dependence arises from the rapid decay of the magnetic field gradient generated by the permanent magnet. For a dipole-like magnetic source, both the magnetic field and, more importantly, the magnetic driving force decrease rapidly with increasing distance, approximately following an inverse-cube (1/r^3^) relationship. Consequently, magnetic actuation becomes increasingly ineffective as the target membrane is located farther from the magnet. Therefore, the cumulative transport profiles therefore reflect an initial magnetically driven enhancement that weakens with increasing separation distance. These results demonstrate that magnetic-field-assisted nanoparticle transport is highly dependent on magnet proximity and highlight a fundamental limitation of magnetic actuation alone for drug delivery into deeper cochlear structures, motivating the exploration of complementary photothermal and mechanical enhancement approaches.

**Figure 6:**
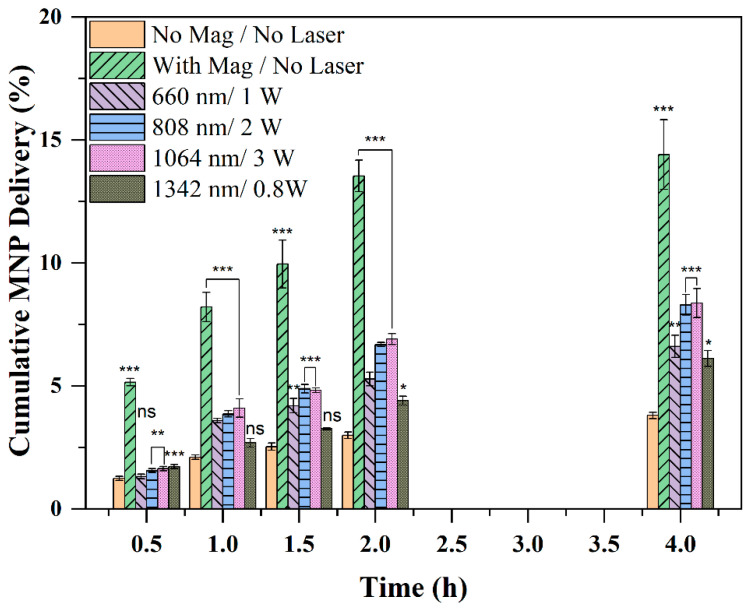
Effect of laser-induced photothermal stimulation on nanoparticle transport across the SIS membrane. Cumulative transport of Fe_3_O_4_ nanoparticles across the SIS membrane under laser irradiation at different wavelengths and power levels. Photothermal stimulation significantly enhanced nanoparticle delivery compared with passive transport, with the greatest transport observed for the 1064 nm/3 W and 808 nm/2 W conditions. Transport enhancement correlated with the photothermal heating response of the nanoparticle suspensions. Data are presented as mean ± SD from *n* = 3 independent membrane/chamber assemblies per condition. Statistical significance was determined at each time point using one-way ANOVA followed by Tukey’s post hoc test compared with the no-magnet control. * *p* < 0.05, ** *p* < 0.01, *** *p* < 0.001, and ns indicates not significant.

Photothermal transport experiments were conducted to investigate whether laser-induced heating could further enhance nanoparticle transport across the membrane without magnetic field. In Figure 6 is CMDP obtained under laser irradiation using the experimental configuration illustrated in Figure 3b. Compared to passive transport, all laser-treated groups exhibited substantially enhanced nanoparticle delivery, although the magnitude of enhancement depended on laser wavelength and power. As a reference, the magnetic-field-assisted transport produced cumulative delivery is shown in this figure, approaching 14.41% after 4 h, while the passive transport remained below 3.49% over the same period. Under photothermal stimulation, CMDP increased significantly beyond the passive condition and, in several cases, approached approximately 8.39% enhancement.

Without a magnetic field, the 1064 nm/3 W and 808 nm/2 W irradiation groups produced the highest CMDP, whereas the 660 nm/1 W condition resulted in a more moderate enhancement. The 1342 nm/0.8 W group exhibited the lowest transport increase among the laser-treated samples. These trends are consistent with the photothermal characterization results shown in Figure 4, where stronger temperature increases generated by the nanoparticles corresponded to greater transport enhancement.

Among the photothermal conditions, 808 nm/2 W and 1064 nm/3 W showed significant enhancement compared with the no-magnet control across all time points, while 680 nm/1 W became significant from 1 h onward. At 4 h, all laser-treated groups showed significantly higher transport than the no-magnet control. The results suggest that laser-induced photothermal heating can effectively facilitate nanoparticle transport across membrane barriers and may provide a practical alternative or complement to magnetic-field-assisted delivery, particularly in applications where magnetic field penetration is limited.

It should be noted that the photothermal heating profiles shown in Figure 4b reached equilibrium temperatures that were generally below 50 °C under the irradiation conditions employed in this study. As shown in Figure 4a, the maximum temperature can be further tuned by adjusting the nanoparticle concentration, providing a practical means of controlling thermal exposure during transport enhancement. For future clinical applications involving NT-3 delivery, photothermal temperatures should be carefully maintained below approximately 42 °C to preserve the structural integrity and biological activity of the neurotrophic protein. This capability to regulate local heating through both laser parameters and nanoparticle concentration may provide an important advantage for balancing transport enhancement with therapeutic safety. While this temperature range was compatible with NT-3 stability, protein stability does not by itself establish safety for native RWM, middle-ear mucosa, or cochlear tissues. The temperatures reported here were measured in the benchtop donor chamber and should not be interpreted as direct measurements of RWM or perilymph temperature in vivo. Native tissue heating would depend on tissue geometry, exposure duration, heat dissipation, perilymph fluid volume, and repeated-dose conditions. Therefore, the present photothermal data should be interpreted as benchtop transport results. Future studies will include thermal dose analysis, temperature-matched controls, membrane histology, and in vivo functional safety assessment.

**Figure 7:**
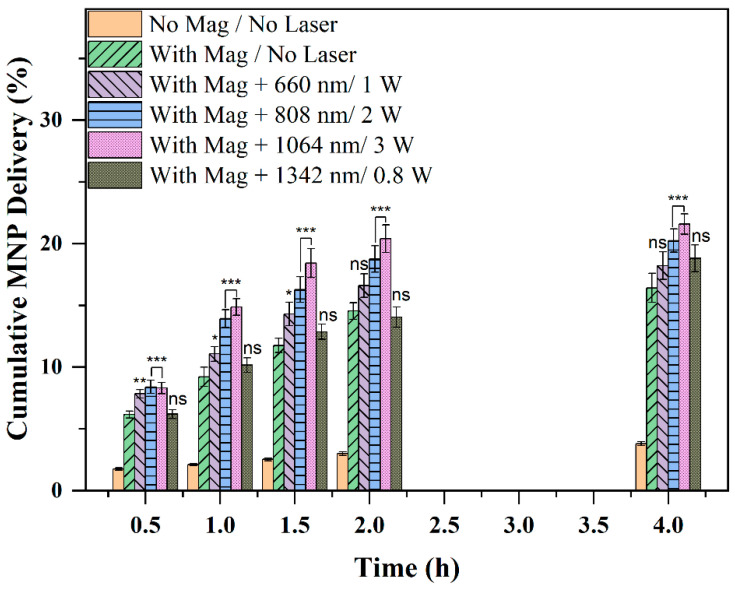
Combined magnetic-field and photothermal enhancement of nanoparticle transport across the SIS membrane. Cumulative transport of Fe_3_O_4_ nanoparticles across the SIS membrane under combined magnetic-field and laser irradiation conditions. All photothermal treatment groups exhibited significantly greater transport than passive diffusion, with the highest cumulative delivery observed for the 1064 nm/3 W and 808 nm/2 W laser conditions. The results demonstrate synergistic enhancement of nanoparticle transport through the combined effects of magnetic guidance and photothermal stimulation. Data are presented as mean ± SD from *n* = 3 independent membrane/chamber assemblies per condition. Statistical significance was determined at each time point using one-way ANOVA followed by Tukey’s post hoc test. Asterisks indicate comparisons between each magnetic–photothermal group and the magnetic-only group: * *p* < 0.05, ** *p* < 0.01, *** *p* < 0.001, and ns indicates not significant.

Combined magnetic-field and photothermal transport experiments were conducted with the set up shown in Figure 2c to evaluate whether laser-induced heating could further enhance magnetic nanoparticle transport across the SIS membrane. As shown in Figure 7, passive transport in the absence of both magnetic field and laser irradiation remained below 3.79% throughout the experimental period. In contrast, the application of magnetic actuation combined with laser irradiation produced substantial increases in CMPD for all laser conditions examined.

CMDP increased steadily with time under all combined magnetic-photothermal conditions, with the greatest enhancement observed for the 1064 nm/3 W and 808 nm/2 W irradiation groups. The 1064 nm laser produced the highest cumulative transport, reaching approximately 21.58% after 4 h, while the 808 nm laser achieved transport levels approaching 20.24%. The 660 nm/1 W and 1342 nm/0.8 W conditions also generated significantly greater delivery than the passive baseline, although their transport enhancement was less pronounced. As can be seen in Figure 7, all magnetic actuations combined with laser irradiations of different wavelengths and powers produced CMPD greater than that with only magnetic field. These trends are consistent with the photothermal heating behavior shown in Figure 4 and suggest that stronger photothermal responses lead to greater transport enhancement. Combined magnetic and photothermal stimulation further enhanced MNP transport compared with magnetic actuation alone, with the degree of enhancement depending on laser wavelength and power. When compared with magnetic actuation alone, 808 nm/2 W and 1064 nm/3 W produced significant additional enhancement across all time points, while 660 nm/1 W showed early enhancement but was not significantly different from magnetic actuation alone at 2 h and 4 h. The 1342 nm/0.8 W condition was not significantly different from magnetic actuation alone. Overall, the results verify that photothermal stimulation provides an additional transport-promoting mechanism when combined with magnetic actuation, resulting in substantially greater nanoparticle delivery than either passive diffusion or magnetic-field-assisted transport alone. The synergistic interaction between magnetic guidance and localized photothermal heating therefore represents a promising strategy for overcoming transport limitations associated with membrane-mediated drug delivery.

**Figure 8:**
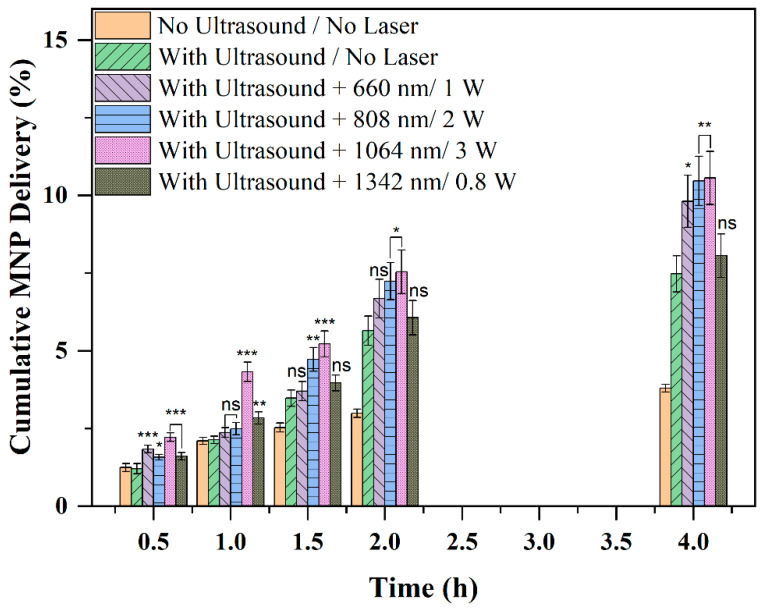
Combined photothermal and ultrasonic enhancement of nanoparticle transport across the SIS membrane in the absence of a magnetic field. Cumulative transport of Fe_3_O_4_ nanoparticles across the SIS membrane under simultaneous laser irradiation and ultrasonication. Passive (zero-field) transport is shown for comparison. Combined photothermal and ultrasonic stimulation significantly increased nanoparticle delivery relative to passive diffusion, with the greatest enhancement observed for the 808 nm and 1064 nm laser conditions. The results demonstrate the feasibility of achieving enhanced membrane transport without magnetic-field assistance through synergistic thermal and mechanical activation. Data are presented as mean ± SD from *n* = 3 independent membrane/chamber assemblies per condition. Statistical significance was determined at each time point using one-way ANOVA followed by Tukey’s post hoc test. Asterisks indicate comparisons between each ultrasound–photothermal group and the ultrasound-only group: * *p* < 0.05, ** *p* < 0.01, *** *p* < 0.001, and ns indicates not significant.

Using the experimental configuration shown in Figure 2d, time-dependent nanoparticle transport studies were performed under combined photothermal and ultrasonic stimulation in the absence of an applied magnetic field. Figure 8 presents CMPD obtained at different time points, together with the passive (zero-field) control for comparison. As shown, the passive transport profile remained relatively low throughout the experimental period, whereas the application of simultaneous laser irradiation and ultrasonication produced a substantial enhancement in nanoparticle transport across the membrane.

Although the enhancement was modest during the initial stages of transport, cumulative delivery increased steadily with time for all treatment groups. In particular, the combined 808 nm and 1064 nm laser irradiation conditions, together with ultrasonication, produced the greatest transport enhancement, reaching cumulative delivery levels exceeding 10.46% after 4 h. These results demonstrate that significant nanoparticle transport can be achieved through the synergistic effects of photothermal heating and ultrasonic stimulation even in the absence of magnetic guidance. The findings are particularly important from a translational perspective, as they suggest a clinically viable strategy for enhancing inner-ear drug delivery without relying on strong external magnetic fields, whose effectiveness diminishes rapidly with distance from the target tissue. Combined ultrasonic and photothermal stimulation enhanced MNP transport compared with ultrasound alone, with the magnitude of enhancement depending on laser wavelength and power. Statistical analysis showed that ultrasound alone significantly increased transport compared with the no-magnet/no-ultrasound control from 1.5 h onward. When compared with ultrasound alone, 808 nm/2 W and 1064 nm/3 W produced significant additional enhancement at multiple time points, while 660 nm/1 W showed significant enhancement at 0.5 h and 4 h. The 1342 nm/0.8 W condition showed early enhancement but was not significantly different from ultrasound alone at 1.5–4 h.

**Figure 9:**
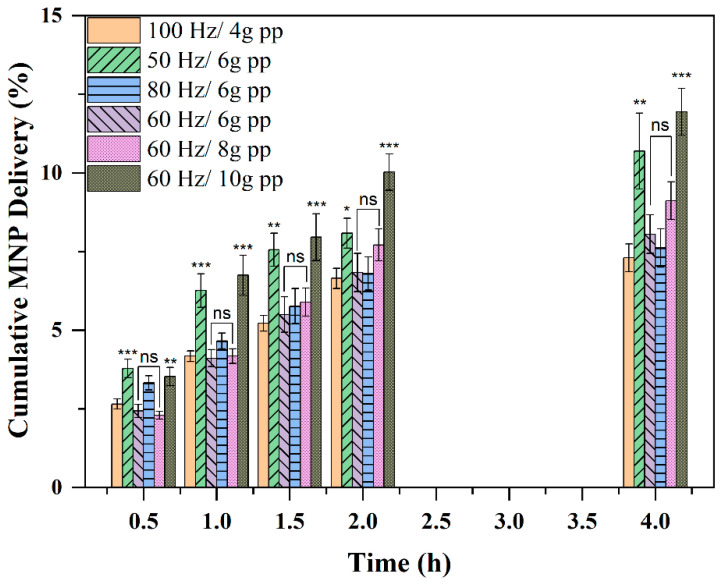
Effect of mechanical vibration on nanoparticle transport across the SIS membrane. Cumulative transport of Fe_3_O_4_ nanoparticles across the SIS membrane under mechanical vibration at different frequencies and amplitudes in the absence of a magnetic field and laser irradiation. Vibration-assisted transport produced significantly greater nanoparticle delivery than passive diffusion, with the highest transport observed at 60 Hz and 10 g peak-to-peak acceleration. The results demonstrate that mechanical oscillation can effectively enhance transmembrane nanoparticle transport through non-magnetic actuation. Data are presented as mean ± SD from *n* = 3 independent membrane/chamber assemblies per condition. Statistical significance was determined at each time point using one-way ANOVA followed by Tukey’s post hoc test. Asterisks indicate comparisons against the 100 Hz/4 g reference condition: * *p* < 0.05, ** *p* < 0.01, *** *p* < 0.001, and ns indicates not significant.

Mechanical vibration-assisted transport experiments were conducted using the setup shown in Figure 2e. Figure 9 presents the cumulative nanoparticle transport profiles across the SIS membrane under mechanical vibration at different frequencies in the absence of a magnetic field and laser irradiation. The vibration applied to the transport setup varied by changing frequency and amplitude (presented as Peak-to-Peak Acceleration denoted as ng pp where n is an integer and g is gravitational constant). As shown in the Figure, nanoparticle delivery increased steadily with time under all vibration conditions, demonstrating that externally applied mechanical oscillation effectively promoted transport across the membrane barrier. Compared with passive diffusion (<3.49%), vibration-assisted transport produced consistently higher cumulative delivery throughout the experimental period, confirming mechanical stimulation as an independent transport-enhancement mechanism. The greatest enhancement was observed at 60 Hz/10g pp, where cumulative transport exceeded 11.93% after 4 h. In contrast, transport at 100 Hz/4g pp reached approximately 7.31%.

These (Figure 9) results indicate that transport efficiency is increased with vibration amplitude and concurrently decreases with vibration frequency. Vibration-enhanced MNP transport depended on both vibration frequency and acceleration. Using 100 Hz/4g pp as the reference condition showed that 50 Hz/6g pp and 60 Hz/10g significantly increased transport at 4 h, while 60 Hz/6g pp, 80 Hz/6g pp, and 60 Hz/8g p were not significantly different from the reference condition at 4 h.

The transport rate enhancement was most pronounced during the early and intermediate stages of the experiment, after which the rate gradually decreased while cumulative delivery continued to rise. This behavior suggests that mechanical vibration enhances transmembrane transport by increasing local fluid motion, reducing boundary-layer resistance, and promoting nanoparticle-membrane interactions during the initial transport period. Overall, these results shows that mechanical vibration alone can significantly improve nanoparticle delivery across semipermeable membranes and may represent a practical non-magnetic, non-thermal strategy for enhancing inner-ear drug transport.

Having established the transport behavior of PEGylated iron oxide nanoparticles under passive, magnetic, photothermal, ultrasonic, and vibrational conditions, dexamethasone-conjugated nanoparticles were evaluated as a therapeutically relevant payload formulation. These studies were designed to determine whether dexamethasone could be successfully incorporated into the PEGylated magnetic carrier and whether the resulting drug-loaded nanoparticles retained their transport capability under passive and externally actuated conditions.

**Figure 10:**
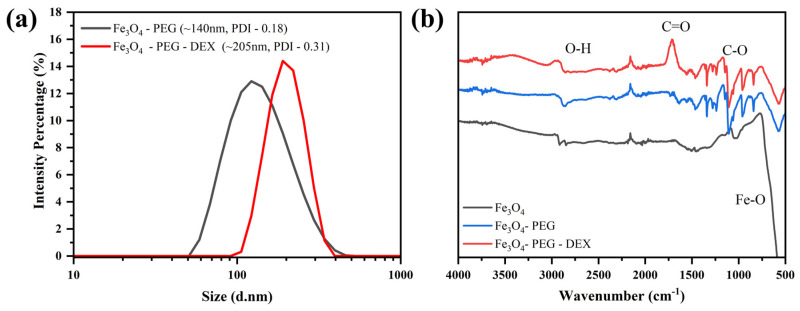
(**a**) DLS intensity distributions of Fe_3_O_4_-PEG and Fe_3_O_4_-PEG-DEX nanoparticles. Dexamethasone conjugation increased the hydrodynamic diameter from approximately 140 nm (PDI = 0.18) for Fe_3_O_4_-PEG PEG to approximately 205 nm (PDI = 0.31) for Fe_3_O_4_-PEG -DEX. (**b**) FTIR spectra of Fe_3_O_4_, Fe_3_O_4_-PEG, and Fe_3_O_4_-PEG-DEX showing retention of the Fe–O band and the appearance of dexamethasone-associated functional groups, including enhanced C=O and C–O features, consistent with successful surface conjugation.

Dexamethasone-conjugated PEGylated iron oxide nanoparticles were synthesized and characterized using dynamic light scattering (DLS), Fourier-transform infrared (FTIR) spectroscopy, and liquid chromatography-mass spectrometry (LC-MS). As shown in Figure 10, dexamethasone conjugation increased the hydrodynamic diameter from approximately 140 nm (PDI = 0.18) for Fe_3_O_4_-PEG nanoparticles to approximately 205 nm (PDI = 0.31) for Fe_3_O_4_-PEG-DEX nanoparticles. FTIR spectra revealed characteristic dexamethasone-associated functional groups, including enhanced carbonyl (C=O) and C–O absorption bands. Dexamethasone conjugation efficiency was estimated by quantifying unreacted free dexamethasone in the post-reaction supernatant using liquid chromatography–mass spectrometry (LC-MS). After the conjugation reaction, Fe_3_O_4_-PEG-DEX nanoparticles were separated from the reaction mixture by magnetic separation/centrifugation, and the supernatant was collected for analysis. The supernatant was diluted as needed and filtered prior to LC-MS injection. Free dexamethasone concentration was determined using an external dexamethasone calibration curve prepared in the same solvent/buffer matrix as the samples. The LC-MS signal intensity or peak area corresponding to dexamethasone was plotted against known dexamethasone concentrations, and the concentration of unreacted dexamethasone in the post-reaction supernatant was calculated from this calibration curve. Together, these results verified successful drug incorporation. Quantification of the conjugated, wash, and supernatant fractions indicated conjugation efficiencies ranging from 45% to 59%, demonstrating effective loading of dexamethasone onto the PEGylated magnetic carrier.

Following confirmation of drug loading, transport studies were performed to evaluate membrane passage of the dexamethasone-conjugated formulation. Passive transport experiments were first conducted using free dexamethasone, unconjugated Fe_3_O_4_-PEG nanoparticles, and Fe_3_O_4_-PEG-DEX nanoparticles as comparative controls. Externally actuated transport studies were subsequently performed under photothermal, magnetic + photothermal, ultrasound + photothermal, and vibration-assisted conditions using the optimized parameters identified from previous experiments. For transport experiments, receiver-chamber samples were quantified using the Ferene-S iron assay for conjugated iron oxide nanoparticles. This assay measures Fe_3_O_4_-associated iron and therefore reflects transport of the Fe_3_O_4_-PEG-DEX nanoparticle carrier. The Ferene-S assay does not directly quantify dexamethasone and does not distinguish free dexamethasone from nanoparticle-conjugated dexamethasone in the receiver chamber. The same analytical approach used for quantification of iron oxide nanoparticles was also applied for quantification of dexamethasone-coated iron oxide nanoparticles.

**Figure 11:**
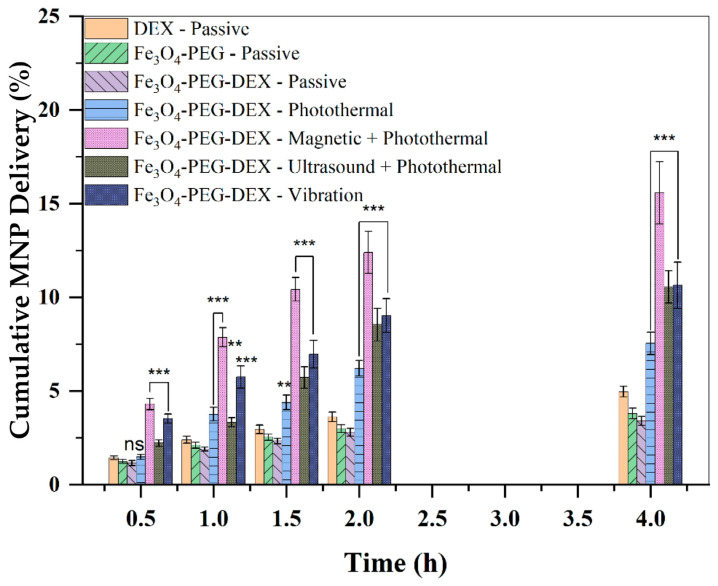
Passive and actuation-assisted transport of dexamethasone-conjugated PEGylated iron oxide nanoparticles across the SIS membrane. Cumulative delivery was compared for free dexamethasone under passive conditions, unconjugated Fe_3_O_4_-PEG nanoparticles under passive conditions, and Fe_3_O_4_-PEG-DEX nanoparticles under passive, photothermal, magnetic + photothermal, ultrasound + photothermal, and vibration-assisted conditions over 4 h. External actuation increased delivery of the dexamethasone-conjugated formulation relative to passive transport, with magnetic + photothermal treatment producing the highest cumulative delivery. Fe_3_O_4_-PEG-DEX transport was quantified using the Ferene-S iron assay and is reported as nanoparticle-associated iron transport. Data are presented as mean ± SD from *n* = 3 independent membrane/chamber assemblies per condition. Statistical significance was determined at each time point using one-way ANOVA followed by Tukey’s post hoc test. Asterisks indicate comparisons against the passive Fe_3_O_4_-PEG-DEX group: * *p* < 0.05, ** *p* < 0.01, *** *p* < 0.001, and ns indicates not significant.

As shown in Figure 11, dexamethasone loading did not eliminate membrane transport and the drug-loaded nanoparticles remained highly responsive to external stimulation. Under passive conditions, cumulative delivery at 4 h reached approximately 4.85% for free dexamethasone, 3.79% for Fe_3_O_4_-PEG nanoparticles, and 3.35% for Fe_3_O_4_-PEG-DEX nanoparticles. Application of external actuation substantially increased delivery of the drug-loaded formulation. Photothermal stimulation increased cumulative transport to approximately 7.69%, while vibration and ultrasound + photothermal treatment produced cumulative deliveries of approximately 10.64% and 10.32%, respectively. The highest transport was achieved under combined magnetic and photothermal stimulation, reaching 15.58% at 4 h, corresponding to an approximately 4.7-fold increase relative to passive Fe_3_O_4_-PEG-DEX transport. Multimodal actuation significantly enhanced the transport of Fe_3_O_4_-PEG-DEX nanoparticles compared with passive Fe_3_O_4_-PEG-DEX transport. Statistical analysis showed that magnetic + photothermal, ultrasound + photothermal, and vibration groups were significantly higher than passive Fe_3_O_4_-PEG-DEX transport across all time points. Photothermal stimulation alone became significantly higher than passive Fe_3_O_4_-PEG-DEX transport from 1 h onward.

These results shows that dexamethasone incorporation did not compromise the responsiveness of the PEGylated iron oxide carrier to magnetic, thermal, or mechanical stimulation. More importantly, the findings extend the multimodal transport platform beyond nanoparticle transport alone and establish its applicability to therapeutically loaded formulations. The ability to achieve substantial enhancement of dexamethasone delivery through externally applied physical fields supports the potential of this platform for controlled and targeted drug delivery across biological membrane barriers.

## 4. Discussion

Magnetically assisted transport of magnetic nanoparticles (MNPs) has been extensively investigated as a strategy for enhancing local drug delivery to the inner ear. Previous studies have demonstrated that externally applied magnetic field gradients can significantly increase MNP transport across the RWM, providing a means to overcome the limitations of passive diffusion and improve cochlear drug access. The present study further confirms the effectiveness of magnetic-field-assisted transport, with cumulative MNP delivery increasing substantially compared with passive conditions. These findings are consistent with earlier reports showing that superparamagnetic nanoparticles can be guided across biological membranes through externally applied magnetic forces.

Despite this promise, the results also reveal a fundamental limitation of magnetic-field-driven transport. As shown in Figure 5, MNP transport decreased sharply as the distance between the magnet and membrane increased. Beyond approximately 2 cm, the enhancement effect diminished rapidly, and, at larger separations, the transport profile approached that of passive diffusion. This behavior originates from the physics of magnetic dipole fields, where magnetic flux density decreases approximately as 1/r^3^ with distance from the magnet, while the magnetic force acting on nanoparticles depends on the magnetic field gradient and therefore decays even more rapidly. Consequently, although magnetic guidance is highly effective in benchtop systems where magnets can be positioned close to the membrane, its clinical utility becomes increasingly challenging for cochlear targets located several centimeters from the skin surface.

To address this limitation, alternative actuation modalities capable of enhancing MNP transport without relying exclusively on magnetic field gradients were investigated. Photothermal stimulation, ultrasonication, and mechanical vibration each significantly increased nanoparticle transport across the membrane. Photothermal enhancement was particularly effective under 808 and 1064 nm irradiation, producing substantial increases in cumulative delivery while maintaining temperatures within a range compatible with thermally sensitive biologics. Ultrasonic stimulation enhanced transport even in the absence of magnetic fields, while mechanical vibration alone produced cumulative transport rates exceeding 10% under optimized conditions. These findings confirm that multiple external energy sources can be utilized to facilitate transmembrane nanoparticle transport and may provide clinically practical alternatives to magnetic-field-only approaches. A limitation of the present study is that histological, viability, and functional safety assessments were not performed after photothermal actuation. Therefore, although photothermal stimulation enhanced nanoparticle transport in the benchtop system, additional RWM tissue studies and in vivo safety testing are required before translational application. Although passive nanoparticle transport was used as the primary baseline in this study, additional mechanistic controls would further strengthen the interpretation of actuation-specific effects. Additionally, because nanoparticle transport was quantified using the Ferene-S iron assay, no-nanoparticle actuation controls would not produce a directly comparable iron-based signal. Such control groups would require a separate tracer-based assay to evaluate membrane leakage or nonspecific permeability changes. Future studies could therefore incorporate additional membrane-integrity assays, such as fluorescent dye leakage, electrical resistance measurements, and temperature-matched heating controls, to further distinguish nanoparticle-mediated transport enhancement from nonspecific membrane permeability changes caused by heating, ultrasound, or vibration.

The observed transport enhancement can be understood through several complementary physical mechanisms. Under magnetic actuation, nanoparticles experience a directional magnetophoretic force that drives transport toward the membrane. Photothermal stimulation increases molecular mobility, enhances Brownian motion, reduces fluid viscosity, and may transiently increase membrane permeability. Ultrasonic stimulation introduces acoustic streaming, microconvection, and oscillatory pressure gradients that reduce concentration polarization near the membrane surface and promote nanoparticle penetration through membrane pores. Mechanical vibration produces similar effects by disrupting stagnant boundary layers and increasing particle-membrane interactions. In each case, the externally applied field supplies additional energy to overcome transport resistance imposed by the membrane barrier.

An important observation from this study is that combinations of external stimuli frequently produced greater MNP transport than any individual modality alone. Combined magnetic and photothermal actuation yielded the highest cumulative transport levels observed for both unconjugated and dexamethasone-loaded nanoparticles. This behavior suggests that transport is governed by several coupled mechanisms acting simultaneously, including magnetophoresis, diffusion, thermally activated transport, and mechanically induced convection. The ability to combine these mechanisms provides a flexible engineering framework for optimizing delivery performance while reducing dependence on any single actuation source.

The dexamethasone-conjugation studies further demonstrate the translational potential of the platform. Dexamethasone was successfully incorporated into PEGylated Fe_3_O_4_ nanoparticles with conjugation efficiencies ranging from 45% to 59%, while retaining the carrier’s responsiveness to external actuation. Although drug loading increased hydrodynamic diameter, the resulting Fe_3_O_4_-PEG-DEX nanoparticles remained capable of crossing the membrane barrier and exhibited substantial enhancement under photothermal, ultrasonic, vibrational, and magnetic-photothermal conditions. Notably, combined magnetic and photothermal stimulation increased dexamethasone-loaded nanoparticle delivery by nearly five-fold relative to passive transport. These findings indicate that therapeutic payload incorporation does not compromise the effectiveness of the multimodal transport strategy and support the use of PEGylated magnetic nanoparticles as both transport vehicles and drug carriers. A limitation of this study is that dexamethasone transport in the receiver chamber was not quantified independently from nanoparticle carrier transport. The Ferene-S iron assay is appropriate for quantifying Fe_3_O_4_-PEG-DEX nanoparticle carrier transport, but it does not directly measure dexamethasone or distinguish free dexamethasone from nanoparticle-conjugated dexamethasone. Direct dexamethasone-specific quantification would require additional extraction or release steps to recover nanoparticle-bound dexamethasone. Future studies will combine Ferene-S iron quantification with dexamethasone ELISA and/or LC-MS to separately evaluate nanoparticle carrier transport, dexamethasone release, and total dexamethasone delivery.

The use of porcine SIS in this study should be interpreted as a controlled acellular screening model rather than a direct biological substitute for native RWM. This distinction is supported by the benchtop RWM study by Goyal et al. [9], which compared SPION transport across acellular SIS, fixed guinea pig RWM, and fresh guinea pig RWM. In that study, SIS served as a semiporous acellular membrane, whereas fresh guinea pig RWM exhibited a trilayer structure consisting of outer epithelium, connective tissue, and inner epithelium. Magnetic-field-assisted transport increased SPION delivery across SIS, fixed RWM, and fresh RWM, but the magnitude and kinetics differed by membrane type. These differences likely reflect the native cellular architecture, tight junctions, extracellular matrix composition, and possible active cellular transport pathways present in RWM but absent in SIS. Therefore, the present SIS-based results should be interpreted as controlled benchtop screening data for comparing nanoparticle formulations and actuation strategies, rather than as a direct quantitative prediction of native RWM transport. Future studies using fresh RWM specimens and in vivo models will be necessary to validate the translational relevance of the observed transport trends.

Several future directions emerge from this work. First, the transport studies reported here were performed primarily using SIS membranes as a controlled semipermeable model system. Validation using native guinea pig and human RWM tissues will be necessary to establish physiological relevance and determine the extent to which the observed enhancement mechanisms translate to biological membranes. Second, optimization of multimodal actuation protocols may further improve transport efficiency while maintaining safe thermal and mechanical exposure limits for delicate cochlear structures. Third, incorporation of additional therapeutic payloads, including neurotrophic factors such as NT-3 and BDNF, will enable direct evaluation of biologically relevant treatment strategies for hearing restoration. Finally, alternative magnetic excitation approaches, including alternating magnetic fields capable of inducing Brownian and Néel relaxation, may provide additional enhancement through localized mechanical motion and mild heating. Together, these developments could lead to clinically viable nanoparticle delivery platforms that utilize coordinated magnetic, photothermal, ultrasonic, and vibrational actuation to overcome the transport barriers that currently limit local inner-ear therapy.

## 5. Conclusions

A multimodal nanoparticle transport platform was developed and evaluated using PEGylated Fe_3_O_4_ nanoparticles transported across semipermeable membrane barriers under passive, magnetic, photothermal, ultrasonic, and vibrational actuation conditions. The magnetic-field-assisted transport significantly enhanced nanoparticle delivery relative to passive diffusion; however, the enhancement decreased rapidly with increasing magnet-to-membrane distance because of the strong distance dependence of magnetic field gradients. These results highlight an important limitation of magnetic guidance alone for clinically relevant inner-ear drug delivery applications.

To overcome this limitation, alternative external actuation modalities were investigated. Photothermal stimulation, ultrasonication, and mechanical vibration each enhanced nanoparticle transport across the membrane, while combined actuation strategies produced the greatest transport efficiencies. In particular, magnetic-photothermal stimulation generated the highest cumulative nanoparticle delivery, indicating that multiple transport mechanisms can act synergistically to overcome membrane transport resistance.

The PEGylated Fe_3_O_4_ platform was further extended to a therapeutically relevant formulation through dexamethasone conjugation. Characterization studies confirmed successful drug loading with conjugation efficiencies of 45–59%, and the resulting Fe_3_O_4_-PEG-DEX nanoparticles retained responsiveness to magnetic, thermal, ultrasonic, and vibrational stimulation. The combined magnetic-photothermal actuation produced the highest dexamethasone delivery, demonstrating that therapeutic payload incorporation does not compromise transport enhancement.

Overall, these findings establish that multimodal external actuation provides a practical strategy for enhancing nanoparticle-mediated drug transport beyond the limitations of magnetic guidance alone. The ability to combine magnetic, photothermal, ultrasonic, and vibrational fields offers a flexible engineering framework for improving delivery of therapeutic agents across biological membrane barriers and supports future development of clinically translatable approaches for local inner-ear drug delivery and hearing restoration therapies.

## Data Availability

Data is available in a publicly accessible repository.

## Supplementary Materials

The following supporting information can be downloaded at https://www.mdpi.com/article/10.3390/bioengineering13070834/s1: Figure S1: Normalized photothermal temperature response of laser wavelengths used for transport experiments. Temperature rise was recorded over time for 660 nm/1 W, 808 nm/2 W, 1064 nm/3 W, and 1342 nm/0.8 W laser irradiation. Because laser power and beam diameter varied among wavelengths, the temperature increase was normalized to laser power and beam area as (ΔT(t)/(PA)), where (ΔT(t)) is the temperature increase at time (t), (P) is laser power, and (A) is beam area. This normalization allowed comparison of relative photothermal response across wavelengths despite differences in delivered power and beam size; Table S1. Actuation parameters used for photothermal, magnetic, ultrasound, and vibration-assisted transport experiments; Table S2. Comparison of Porcine SIS and native RWM as transport barriers.
