# Supplementary material for: Multimodal Magnetic, Photothermal, Ultrasonic, and Vibrational Actuation of Drug-Loaded Superparamagnetic Iron Oxide Nanoparticles for Enhanced Transport Across Semipermeable Membranes
Source: Bioengineering (Basel). 2026 Jul 21;13(7):834. doi: 10.3390/bioengineering13070834 (PMC13405497; doi:10.3390/bioengineering13070834)
Supplement: Supplementary file 1 [file bioengineering-13-00834-s001.zip › bioengineering-4412643-supplementary.pdf]

Supplementary Information

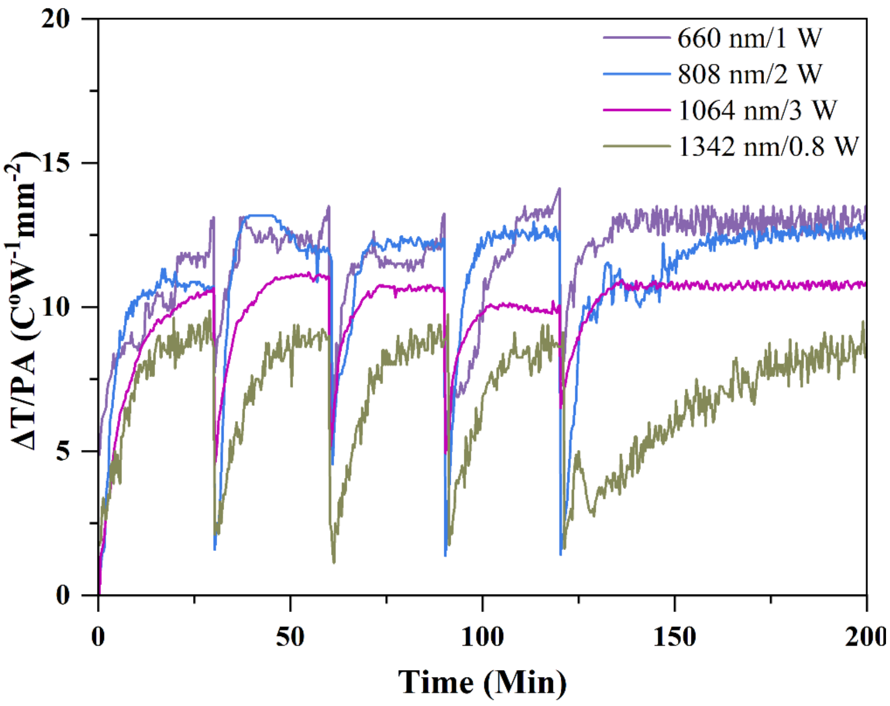

Figure S1. Normalized photothermal temperature response of laser wavelengths used for transport experiments. Temperature rise was recorded over time for 660 nm/1 W, 808 nm/2 W, 1064 nm/3 W, and 1342 nm/0.8 W laser irradiation. Because laser power and beam diameter varied among wavelengths, the temperature increase was normalized to laser power and beam area as (ΔT(t)/(PA)), where (ΔT(t)) is the temperature increase at time (t), (P) is laser power, and (A) is beam area. This normalization allowed comparison of relative photothermal response across wavelengths despite differences in delivered power and beam size.

Table S1. Actuation parameters used for photothermal, magnetic, ultrasound, and vibration-assisted transport experiments

**Actuation mode**	**Main setting**	**Measured/calculated value at sample**	**Geometry/alignment**
Phototherm al 660 nm	1W	Beam area: 0.815 mm^2; Max ΔT: 11 °C	Beam centered over membrane region; - normal to membrane plane
Phototherm al 808 nm	2 W	Beam diameter: 0.760 mm^2; Max ΔT: 19 °C	Same as above
Phototherm al 1064 nm	3 W	Beam diameter: 0.933 mm^2; Max ΔT: 28 °C	Same as above
Phototherm al 1342 nm	0.8 W	Beam diameter: 1.417 mm^2; Max ΔT: 8.5°C	Same as above
Magnetic	Static field	Field at membrane: 0.488T	Pole face parallel to membrane; magnet axis normal to

			membrane; centered at membrane
Ultrasound	40 kHz	Bath temperature: 30-40 °C Depending on the duration	Chamber fixed at consistent location in bath fixed to the bottom surface of ultrasonicate
Vibration	[50–100 Hz], [4–10 g pp]	Acceleration measured at DCRTS surface (identical to the acceleration at membrane)	Chamber fixed to platform; vibration direction normal to membrane

Table S2. Comparison of Porcine SIS and native RWM as transport barriers

**Feature**	**Porcine SIS membrane**	**Native round window membrane**
Biological structure	Acellular extracellular matrix membrane	Trilayer biological membrane
Cellularity	No viable epithelial cells	Outer epithelial layer, connective tissue layer, and inner epithelial layer
Tight junctions	Absent	Present, especially in the epithelial layer facing the middle ear
Active transport	Absent	Possible active transcellular pathways, including endocytosis/macropinocytosis
ECM composition	Collagen-rich decellularized matrix	Native connective tissue with epithelial and extracellular matrix components
Thickness	Depends on commercial SIS preparation and hydration state	Species-dependent; guinea pig RWM is approximately 15–25 µm thick
Transport mechanism	Primarily passive diffusion, steric restriction, and externally assisted transport	Passive diffusion plus possible active cellular transport and epithelial barrier effects
Reproducibility	Higher experimental consistency and easier mounting	More biologically relevant but fragile and variable
Translational role	Useful benchtop screening model for comparing nanoparticle formulations and actuation modes	More physiologically relevant validation model for cochlear delivery
Limitation	Does not reproduce native cellular architecture or active transport	Limited availability, fragile tissue, more variability
